# *In Vitro* Antimicrobial Potential of the Lichen *Parmotrema* sp. Extracts against Various Pathogens

**Published:** 2013-07

**Authors:** Ritika Chauhan, Jayanthi Abraham

**Affiliations:** 1 Microbial Biotechnology Laboratory, School of Biosciences and Technology, VIT University, Vellore-632014, Tamil Nadu, India.

**Keywords:** Antimicrobial agents, Kirby-Bauer Method, Lichens, Multi-drug resistance

## Abstract

***Objective(s):*** The ongoing increasing antibiotic resistance is one of the biggest challenges faced by global public health. The perennial need for new antimicrobials against a background of increasing antibiotic resistance in pathogenic and opportunistic microorganisms obliges the scientific community to constantly develop new drugs and antimicrobial agents. Lichens are known prolific sources of natural antimicrobial drugs and biologically active natural products. This study was aimed to explore *in vitro* antimicrobial activity of lichen* Parmotrema *sp.

***Material and Methods:*** The methanol and aqueous extracts of lichen *Parmotrema *sp. was extracted using Soxhlet extractor. Antibiotic assessment of methanol and aqueous extracts was done against eight bacterial (*Escherichia coli, Staphylococcus aureus,* *Proteus mirabilis, Salmonella *sp*., Shigella *sp*., Enterococci faecalis, Pseudomonas aeruginosa, Klebsiella pneumoniae,*) clinical pathogens and five plant pathogenic fungal strains (*Aspergillus terreus* strain JAS1, *Scedosporium *sp. JAS1, *Ganoderma *sp. JAS4, *Candida tropicalis* and *Fusarium* sp.) by Kirby-Bauer method.

***Results:*** The methanol lichen *Parmotrema *sp. extract inhibited all the test organisms. The highest antibacterial activity was found against *Pseudomonas aeruginosa* and *Staphylococcus aureus*. The weakest activity was manifested in *Salmonella *sp. and *Scedosporium* sp. JAS1. Strong antifungal effect was found against *Ganoderma* sp. JAS4 and *Fusarium* sp. The aqueous lichen *Parmotrema *sp. extract revealed neither antibacterial nor antifungal activity.

***Conclusion: *** The present study shows that tested lichen *Parmotrema *sp. extracts demonstrated a strong antimicrobial effect. That suggests the active components from methanol extracts of the investigated lichen *Parmotrema *sp. can be used as natural antimicrobial agent against pathogens.

## Introduction

Lichens are symbiotic organisms composed of a fungal partner (mycobiont) in association with one or more photosynthetic partners (photobiont). The photobiont can be green algae, cyanobacteria, or both (1). They usually grow on rocks, non-fertile ground, as well as epiphytes on the trees and leaves (2). Lichens have also, for hundreds of years, been used in many countries as a cure for diseases of humans (3). In many European countries numerous species have been used for treatment of stomach diseases, diabetes, whooping cough, pulmonary tuberculosis, cancer treatment and skin diseases (4). The usage of lichens for many years in the traditional medicine was later justified by numerous researches that confirmed their various biological activities.

Lichens produce secondary metabolites called the “lichen substances," which comprise depsides, depsidones, dibenzofurans, xanthones and terpene derivatives. These metabolites sometimes make more than 30% of the dry mass of thalus (3). Various biological activities of lichens and their metabolites are known, such as antiviral, antibiotic, antitumoral, antiallergic, antiherbivoral and they inhibit growth of plants as well as various enzymes (5-6). It has been observed that “Lichens extracts” and “Lichen substances” produce antimicrobial agents (3-4, 7-9) and continuous and uncontrolled use of synthetic drugs has led to the need to find new preparations of natural product drugs (3).

Bioactive natural products have more beneficial effects on organism when compared to synthetic drugs. By considering the multi-drug resistant to pathogens towards infectious diseases, this study was carried out to screen for antimicrobial agents from natural origin.

**Table 1:** Antibacterial activity of lichen *Parmotrema *sp. against the organisms tested by well-diffusion assay

Clinical isolates	Methanol extract of lichen *Parmotrema *sp. (50 mg/ml)											
	25 µl (mm) 50 µl (mm) 75 µl (mm) 100 µl (mm)											
*Staphylococcus aureus*	ME	AQ	S	ME	AQ	S	ME	AQ	S	ME	AQ	S
	17.66±4.78	-	-	22.66±4.49	-	-	26.00±3.26	-	18.0±00	26.66±3.85	-	20±0.0
*Escherichia coli*	6.66±2.42	-	-	18.66±3.29	-	-	22.66±1.24	-		24.00±0.81	-	15±0.0
*Proteus mirabilis*	-	-	-	-	-	-	-	-	-	14.66±0.47	-	15±0.0
*Shigella *sp.	9.33±0.47	-	-	11.00±0.81	-	-	10.66±1.69	-	-	15.00±0.81	-	15±0.0
*Salmonella *sp.	-	-	-	-	-	-		-	-	12.66±0.47	-	15±0.0
*Enterococci faecalis*	10.33±0.47	-	-	11.33±0.94	-	-	13.66±2.05	-	-	16.66±1.24	-	15±0.0
*Pseudomonas aeruginosa*	11.33±8.05	-	-	18.00±1.41	-		21.33±0.47	-	-	24.33±0.47	-	20±0.0
*Klebsiella pneumoniae*	-	-	-	20.00±0.81	-	-	22.00±1.41			23.66±1.24		24±0.0

Me: methanol extract; AQ: aqueous extract; S: standard

## Materials and Methods

***Sample Collection***

Lichen *Parmotrema *sp. was collected from Kodaikanal forest, India in September, 2011. Samples were dried at room temperature for 72 hr. Lichens were ground finely in mortar and pestle for further experiments.

***Preparation of Lichen*** ***Parmotrema*** **sp** ***extracts***

Finely ground thallus of lichen *Parmotrema *sp. (30 g) was extracted using methanol and water separately in a Soxhlet extractor not exceeding the boiling point of the solvent. The obtained extracts were filtered through Whattman Filter Paper No.1 and then concentrated under reduced atmospheric pressure. The dry extracts were stored at -20°C. The extracts were further dissolved in 5% dimethyl sulphoxide (DMSO) for further experimental assays.

***Test organisms***

The bacteria and fungi used as the test organisms in this study were *Escherichia coli, Staphylococcus aureus,* *Proteus mirabilis, Salmonella *sp*., Shigella *sp*., Enterococci faecalis, Pseudomonas aeruginosa, Klebsiella pneumoniae, Candida tropicalis* and *Fusarium* sp. All the clinical isolates were acquired from Thanjavur Medical College, Thanjavur, Tamil Nadu, India. The fungi used as test organisms were *Aspergillus terreus* strain JAS1, *Scedosporium *sp. JAS1, *Ganoderma *sp. JAS4 were procured from Microbial Biotechnology Lab, SBST, VIT University, Vellore, India. Bacterial and fungal clinical isolates were maintained on Nutrient agar and Potato Dextrose Agar respectively.

***Antibiogram***

The multi-drug resistant bacterial test pathogens were screened against standard antibiotic disc, vancomycin (30 mcg/disc), tigecycline (15 mcg/disc), erythromycin (15 mcg/disc), ciprofloxacin (30 mcg/disc), penicillin (10 mcg/disc), ofloxacin (5 mcg/disc) and fungal isolates were screened against flucanazole (25 mcg/disc) and voriconazole (5 mcg/disc). Antibiotic sensitivity test was performed by Kirby-Bauer method on Muller-Hinton agar plates (10). Bacterial test pathogens were lawn cultured on Muller-Hinton agar plates using sterile cotton swabs. The standard antibiotic discs were placed on it using sterile forceps. Plates were incubated at 37°C for 24-48 hr and were observed for zone of inhibition. Fungal test pathogens were seeded into Potato Dextrose Agar petridishes, antibiotics disc was placed on it using sterile forceps. Plates were incubated at 30°C for 5 days and were observed for zone of inhibition.

***Antimicrobial assay***

The sensitivity of microorganisms to methanol and aqueous extracts were tested by measuring the zone of inhibition of given concentration of lichen *Parmotrema *sp. extracts by the well-diffusion method. Clinical bacterial isolates were swabbed onto Muller-Hinton agar plates. Four wells were punctured onto the agar plate. 50 mg/ml of dry lichens extract with different concentrations (25 µl, 50 µl, 75 µl and 100 µl) were loaded into the wells. The petriplates were incubated for 24 hr, and the zone of inhibition was measured around the wells. 

For antifungal activity, the appropriate fungal test pathogens were seeded in potato dextrose agar (PDA) in petridishes (11). Paper disks of 6 mm in diameter were laid on the inoculated test organism after being soaked with 15 µl of the lichen extract of 50 mg/ml, antimicrobial activity was determined by measuring the zone of inhibition around the disk. Streptomycin was used as positive control of the growth in bacteria and flucanazole in case of fungi. DMSO (dimethyl sulfoxide) solution was used as negative control of the influence of solvents. All the experiments were performed in triplicates.

**Table 2:** Antifungal activity of lichen *Parmotrema *sp. against the organisms tested by disc-diffusion method

Fungal Test organisms	Zone of Inhibition (mm)		
*Candida tropicalis *	ME	AQ	F
	20±0.81	-	-
*Fusarium *sp.	21.33±0.471	-	-
*Scedosporium *sp*. *JAS1	-	-	-
*Ganoderma *sp*. *JAS4	21.00±0.00	-	-
*Aspergillus terreus* strain JAS1	16±0.81	-	-

ME= methanol extracts, AQ= aqueous extracts, F= Flucanazole, mm= millimeters

**Figure 1:**
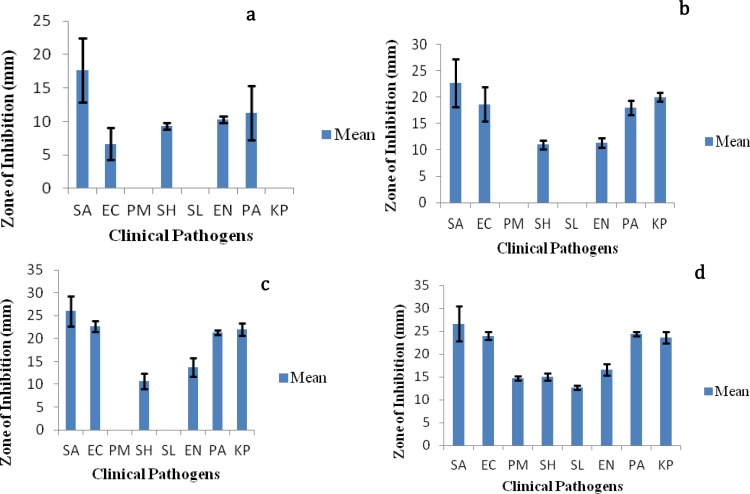
Antibacterial activity of (25, 50, 75, 100 µl) methanol extract of lichen *Parmotrema *sp. SA= *Staphylococcus aureus*, EC=*Escherichia coli*, PM= *Proteus mirabilis*, SH=*Shigella* sp., SL=*Salmonella* sp., EN=*Enterococci faecalis*, PA=*Pseudomonas aeruginosa*, KP=*Klebsiella pneumoniae*

## Results

***Antibiogram***

The test pathogens screened against standard antibiotic disc did not show zone of inhibition against vancomycin (30 mcg/disc), tigecycline (15 mcg/disc), erythromycin (15 mcg/disc), ofloxacin (5 mcg/disc), flucanazole (25 mcg/disc) and voricanazole (5 mcg/disc).

***Antimicrobial assay***

The antimicrobial activity of methanol and aqueous extracts of lichen* Parmotrema *sp. against the test microorganisms was estimated based on presence or absence of inhibitory zones and their diameter value of the extract. Antibacterial activity of lichen extracts is shown in Table 1 and antifungal activity in Table 2.

Antibacterial activity of lichen *Parmotrema *sp. was manifested by aqueous and methanol extracts. They inhibited all the tested species of bacteria except *Salmonella *sp. which showed a low zone of inhibition. Antifungal activity was manifested against five fungal pathogens, in which *Scedosporium* sp turned out to be resistant. The aqueous extracts manifested neither antibacterial nor antifungal activity. The weakest activity of methanol extracts of lichen *Parmotrema *sp. was manifested against *Salmonella *sp. (12 mm) and *Aspergillus terreus* JAS1 (16 mm) and the largest zone of inhibition against *Staph. aureus* (26 mm), *P. aeruginosa* (24 mm), *Ganoderma *sp. JAS4 (21 mm) and *Fusarium *sp. (21 mm) with 50 mg/ml concentration. The different concentration (25 µl, 50 µl, 75 µl and 100 µl) of methanolic extract of lichen *Parmotrema *sp. exhibiting antimicrobial activity has been represented graphically (Figure 1), which further demonstrates the same. 

DMSO was used as a negative control, when tested in applied concentration of lichen *Parmotrema *sp. extract DMSO had no inhibiting effect on the tested organisms. Streptomycin served as the positive control, which inhibited the growth of all the bacteria tested, while flucanazole inhibited none of the fungi.

## Discussion

Antimicrobial potential of lichen *Parmotrema* sp. extract against multi-drug resistant pathogens has been presented in this work. The results obtained in this study showed strong antimicrobial action on the test pathogens, which usually depends on the species of lichen, the type of extracting solvent used and the concentration of lichen extract. Similar differences were observed by other authors (9, 12-14). Aqueous extracts manifested no activity in relation to the microorganisms tested. Land and Lundstrom (15) reported that aqueous extracts of the lichen *Nephroma articum* have no antifungal effects. The reason for weak activity of aqueous extracts is that active substances present in the thalli of lichens are insoluble or poorly soluble in water (16). Gulluce *et al* (8) found that a methanol extracts of the lichen *Parmelia saxatilis* had stronger antibacterial than antifungal activity. Candan *et al* (17) established antimicrobial activity for different extracts of the lichen *Xanthoparmelia pokorny* against bacteria and yeasts, but not against filamentous fungi. The present study indicates that the methanol extracts of lichen *Parmotrema *sp. exhibit strong antibacterial as well as antifungal activity. Lichen *Parmotrema *sp. extracts inhibited all the tested micro-organisms. 

**Figure 2:**
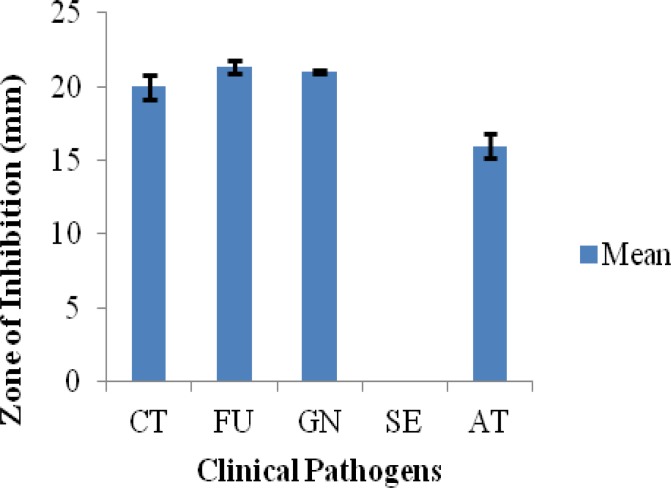
Antifungal activity of 15 µl methanol extract of lichen *Parmotrema *sp. CT=*Candida tropicalis*, FU=*Fusarium* sp., GN=*Ganoderma* sp. JAS4, SE=*Scedosporium* sp JAS1, AT=*Aspergillus terreus* strain JAS1

## Conclusion

The investigated lichen *Parmotrema *sp. extracts revealed strong, but varying degree of antimicrobial activity. The antimicrobial action of lichen *Parmotrema *sp. from Kodaikanal forest are probably the consequence of different antimicrobial agents which can be employed in treatment of various diseases caused by these pathogens. This suggests that active components from methanol extracts of the investigated lichen *Parmotrema *sp. can be used as natural antimicrobial agents for possible formulation of new drug to fight against pathogens.
